# Lybatides from *Lycium barbarum* Contain An Unusual Cystine-stapled Helical Peptide Scaffold

**DOI:** 10.1038/s41598-017-05037-1

**Published:** 2017-07-12

**Authors:** Wei Liang Tan, Ka H. Wong, Jian Lei, Naoki Sakai, Hong Wei Tan, Rolf Hilgenfeld, James P. Tam

**Affiliations:** 1 School of Biological Sciences, Nanyang Technological University, Singapore, Singapore; 2 Institute of Biochemistry, Center for Structural and Cell Biology in Medicine, University of Lübeck, Ratzeburger Allee 160, 23562 Lübeck, Germany; 3 German Center for Infection Research (DZIF), Hamburg–Lübeck–Borstel–Riems Site, University of Lübeck, Lübeck, Germany

## Abstract

Cysteine-rich peptides (CRPs) of 2–6 kDa are generally thermally and proteolytically stable because of their multiple cross-bracing disulfide bonds. Here, we report the discovery and characterization of two novel cystine-stapled CRPs, designated lybatide 1 and 2 (lyba1 and lyba2), from the cortex of *Lycium barbarum* root. Lybatides, 32 to 33 amino acids in length, are hyperstable and display a novel disulfide connectivity with a cysteine motif of C-C-C-C-CC-CC which contains two pairs of adjacent cysteines (-CC-CC). X-ray structure analysis revealed the presence of a single cystine-stabilized (α + π)-helix in lyba2, a rare feature of CRPs. Together, our results suggest that lybatides, one of the smallest four-disulfide-constrained plant CRPs, is a new family of CRPs. Additionally, this study provides new insights into the molecular diversity of plant cysteine-rich peptides and the unusual lybatide scaffold could be developed as a useful template for peptide engineering and therapeutic development.

## Introduction

*Lycium barbarum*, belonging to the Solanaceae family, is a deciduous shrub native to southeastern China^1^. It is the plant that produces the popular herb wolfberries or goji berries. However, the cortex of *L*. *barbarum* root ( or DiGuPi in Chinese) is also commonly used as a traditional Chinese medicine for treating chronic low-grade fever, cough, hemoptysis and hematuria, diabetes mellitus and hypertension^1^. A diverse group of secondary metabolites has been isolated from the cortex of *L*. *barbarum* root, including alkaloids, flavonoids and flavone glycosides. In addition, small cyclic peptides, namely licyumins A‒D, with molecular weights <1 kDa, have been shown to inhibit renin and angiotensin-converting enzymes^2^. However, no bioactive peptides >2 kDa have been reported.

Bioactive compounds from medicinal plants have been a source of inspiring structures for drug discovery. A majority of studies focuses on small molecules and secondary metabolites, with few studies focusing on peptides^3, 4^. Even in studies relating to identifying peptides from plants, the focus is largely on small cyclic peptides. This bias is attributed to a general perception that peptides >2 kDa are unstable and readily denatured during a decoction preparation or in the gastrointestinal tract after ingestion. This is generally true for large peptides and proteins with molecular weight >8 kDa. However, cysteine-rich peptides (CRPs) with a molecular range of 2–6 kDa and 3–5 disulfide bonds are tolerant to thermal, chemical and proteolytic degradation^5^. As a group, CRPs in this defined chemical space have great potential as a source of leads and inspiration for developing useful drugs from medicinal plants^6, 7^.

**Figure 1:**
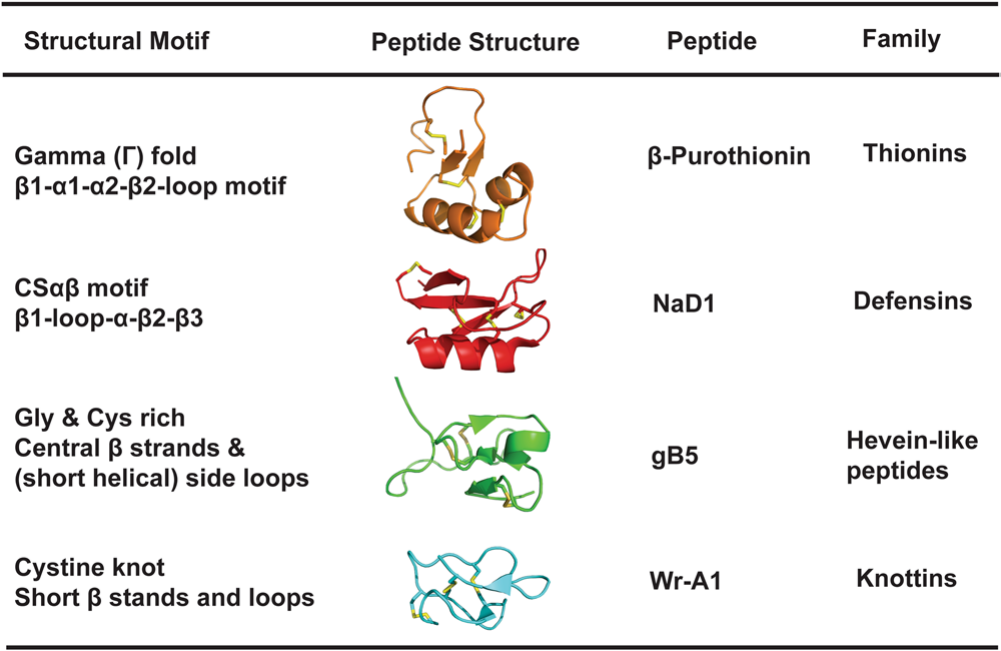
List of major plant CRP families with representative member within the chemical space of 2–6 kDa can be arbitrarily classified into two major groups. Plant thionins and defensins contain α-helix in their structural motif while hevein-like peptides and knottins contains a majority of β-strands. (PDB ID: 1BHP, 4AB0, 5XIV, 2MI9).

Structurally, plant CRPs within the chemical space of 2–6 kDa can be arbitrarily classified into two major groups (Fig. 1). They are the cystine-stabilized α-helical (CSα) peptides and cystine-stabilized β-peptides. The CSα peptides can be found in plant CRPs >40 residues such as plant defensins and plant thionins. Cystine-stabilized β-peptides are found in plant CRPs with <40 residues, such as knottins and hevein-like peptides, and they generally contain short β-strands and no well-formed α-helix^6^. In contrast, CSα peptides contain a dominant cystine-stapled helix with at least three turns in their structure. In addition, their helical conformation is usually stabilized by another well-defined secondary structure such as an α-helix or one or more β-strands. They include cystine-stabilized α/β motif (CSαβ) of plant defensins^8, 9^, and the cystine-stabilized α-α helical motif (CSαα) of thionins^10^.

Here, we report the identification and characterization of two novel cystine-stapled helical CRPs, namely lybatide 1 and 2 (lyba1 and lyba2), from the root bark of *Lycium barbarum*. Differing from CSαβ and CSαα peptides, lybatide2 presents a helix in its structure that is not stabilized by another well-defined secondary structural element as determined by X-ray crystallography. Comparison of its cysteine arrangements, disulfide connectivity and overall three-dimensional structure to other cysteine-rich peptides show that lybatides represent a new family of CRP with an unusual cystine-stabilized helical structure that has not been described in other plant CRPs.

## Results

### Isolation of lyba1 and lyba2 from *L*. *barbarum*

**Figure 2:**
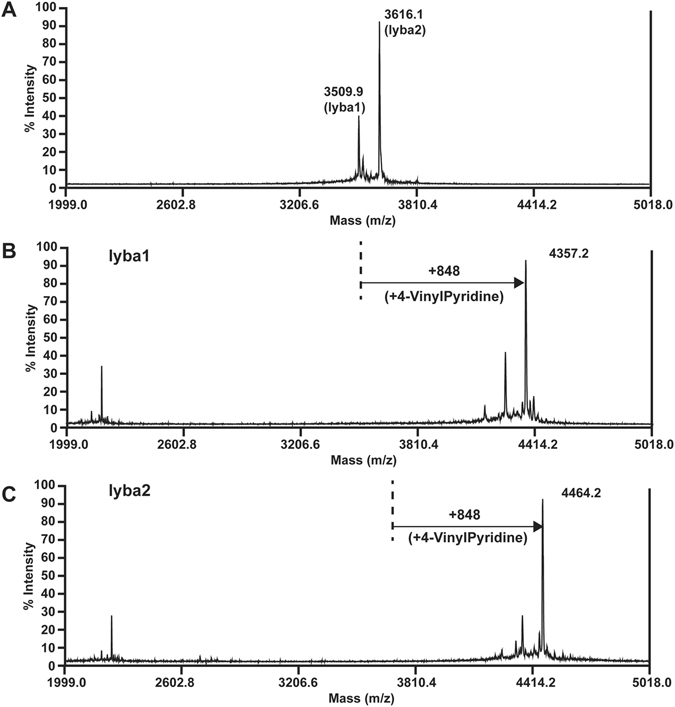
MALDI mass spectrum of lybatides, lyba1 and lyba2. (**A**) Mass spectrum displaying two main peaks at m/z 3509, lyba1, and m/z 3616, lyba2. (**B**) 4-vinylpyridine alkylation of lyba1 after reduction by dithiothreitol, showing a m/z shift of 848. (**C**) 4-vinylpyridine alkylation of lyba2 after reduction by dithiothreitol, showing a m/z shift of 848.

Mass spectrometry-guided screening of an aqueous extract of *L*. *barbarum* root bark revealed the presence of two peptides with m/z 3509 and 3616 Da (Fig. 2A). MALDI-TOF MS analysis of these peptides after reduction with dithiothreitol and *S*-alkylation with 4-vinylpyridine result in a mass shift of 848 Da, indicating the presence of eight cysteine residues in these peptides (Fig. 2B,C). The two peptides, lyba1 and lyba2, were isolated in a scaled-up extraction and purification process. To determine their amino-acid sequences, lyba1 and lyba2 were fully reduced with dithiothreitol and *S*-alkylated with 4-vinylpyridine followed by digestion with trypsin or chymotrypsin. Tryptic digestion of the *S*-alkylated lyba1 generated two fragments with m/z values of 2523 and 1853 Da, while chymotrypsin digestion generated four fragments with m/z values of 1577, 2799, 2490 and 1886 Da. *De novo* sequencing of the digested fragments gave the full sequence of the 32-residue Lyba1 as DSCSEYCSNNSCPYCDGQKLYTLCCINTCCPS (Fig. S1). The process was repeated to determine the sequence of the 33-residue Lyba2 as DSCSEYCSNRCPSCDGQTQTQYTLCCINICCPS (Fig. S2). Assignment of isobaric Ile and Leu was based on homologous expressed sequence tag sequences from the OneKP transcriptome database (www.onekp.com). Alignment of lybatides revealed that they contain eight highly conserved cysteine residues arranged in a unique spacing of C-C-C-C-CC-CC, with a sequence identity of 76.5% between the two. Interestingly, there are two consecutive CC motifs at the C-terminus of the lybatides. Both lyba1 and lyba2 are anionic with a theoretical isoelectric point of 4.03 and are serine-rich, containing five serine residues out of 32 or 33 amino acid residues, respectively.

### Determination of the disulfide connectivity of lyba1

The disulfide connectivity of lybatides was elucidated by a stepwise partial alkylation strategy using a *S*-tagged alkylation of lyba1 with iodoacetamide and N-ethylmaleimide (NEM), followed by MS/MS analysis^5^. Lybatide lyba1 was partially *S*-reduced by TCEP and *S*-alkylated by N-ethylmaleimide to generate a mixture of intermediates containing 1-SS, 2-SS and 3-SS species. The partially *S-*alkylated intermediates were then purified with RP-HPLC. Full reduction with dithiothreitol followed by *S*-alkylated with iodoacetamide, give rise to three major groups of mixed alkylated products. These products were then analyzed by MALDI-TOF MS/MS.

**Figure 3:**
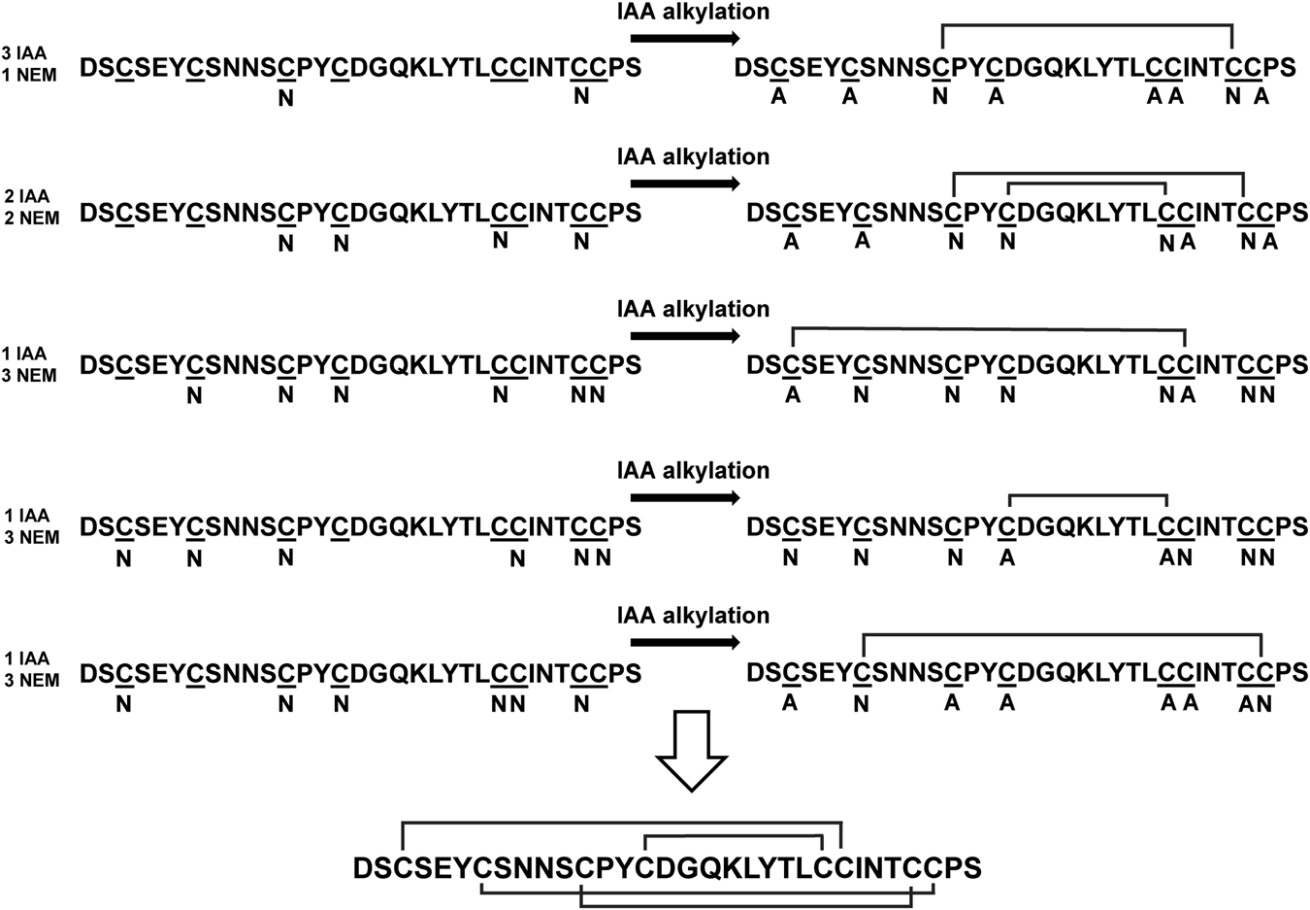
The disulfide structure was elucidated by S-tagged alkylation with iodoacetamide (IAA) and N-ethylmaleimide (NEM) of the peptide using a stepwise partial reduction alkylation strategy followed by MS/MS analysis. **N** indicates cysteine residues alkylated with NEM and **A** indicates cysteine residues alkylated with IAA. Five partially reduced intermediates were alkylated with NEM followed by full reduction by dithiothreitol and S-alkylation with IAA. MS/MS-analysis of the differently S-alkylated peptides allows us to deduce the disulfide connectivity of lyba1.

MS/MS fragmentation patterns of the double *S*-tagged peptides provided clues to determine the cysteine linkages of the peptide by observing which cysteine residues (Fig. 3) were differentially *S-*alkylated by N-ethylmaleimide and iodoacetaminde. This modification produced five differentially *S-*alkylated products, two with a mass of 4109 Da, one with a mass of 4245 Da and two with a mass of 4381 Da, depending on the ratio of N-ethylmaleimide-modified and iodoacetamide-modified cysteines. MS/MS sequencing of the two N-ethylmaleimide-modified peptides sharing a mass of 4109 Da revealed the position of the two N-ethylmaleimide-modified cysteines and established the disulfide connectivity of CysIII–CysVII and CysII–CysVIII. Likewise, the locations of the iodoacetamide-modified cysteines of the two peptides sharing a mass of 4381 Da established the remaining disulfide bonds as CysI–CysVI and CysIV–CysV. Combining the two sets of data led to the complete disulfide connectivity of lyba1 as CysI–CysVI, CysII–CysVIII, CysIII–CysVII and CysIV–CysV.

### X-ray crystal structure of lyba2

**Figure 4:**
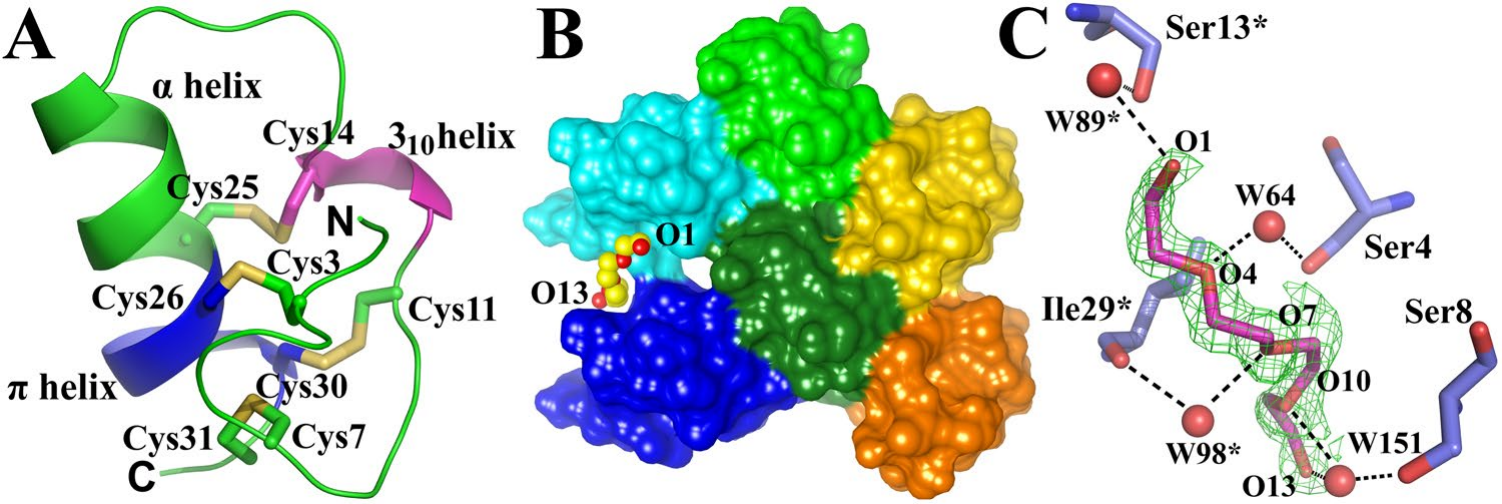
The three-dimensional structure of lyba2. (**A**) Cartoon view of the overall structure. Four disulfide bonds are formed by Cys3–Cys26, Cys7–Cys31, Cys11–Cys30 as well as Cys14–Cys25. The 3_10_ helix and π helix are colored purple and blue, respectively. (**B**) Packing of the 3 copies of lyba2 and their symmetry-mates in the crystal. Molecules A, B and C are colored green, pink and blue, respectively. The polyethylenglycol (PEG) molecule is shown in sphere view. The terminal oxygen atoms of the PEG are labeled. (**C**) Details of the interactions between PEG and lyba2. An Fo-Fc electron density of the PEG molecule (green; contoured at 2.5 σ level) is displayed. Hydrogen bonds between the PEG and lyba2 are indicated as dashed lines. Symmetry-related water molecules and amino-acid residues are labelled by an asterisk (*).

The lyba2 peptide was used to determine the 3D structure because it was more easily crystallized. Its 1.48-Å three-dimensional structure was determined by the sulfur single-wavelength anomalous dispersion (S-SAD) method^11^ (Fig. 4). The final refinement statistics are presented in Table S1. There are three copies of the peptide molecule (named A, B and C) per asymmetric unit (ASU) of the crystal. The root-mean-square deviations (for Cα atoms) between A and B, A and C, and B and C are 0.44, 0.46 and 0.36 Å, respectively. The last residue (Ser 33) of molecule A is undefined due to incomplete electron density. The *R*_*cryst*_ and *R*_*free*_ of the final model are 0.110 and 0.137, respectively.

The crystal structure revealed that lyba2 contains a turn (Ser2‒Tyr6), a one-turn 3_10_ helix (Pro12‒Cys14), and an α helix (Gln19‒Leu24 in molecule A, Gln19–Cys25 in molecules B and C) that extends into a somewhat distorted π helix (Cys25‒Cys30 in molecule A, Cys26‒Cys30 in molecules B and C; Fig. 4A). A π helix is a widened α-helix with 4.1 residues per turn (instead of 3.6) and hydrogen bonds between atoms N (i + 5) and O (i). The four disulfide bonds are formed by Cys3‒Cys26, Cys7‒Cys31, Cys11‒Cys30 and Cys14‒Cys25 according to the X-ray structure (Fig. 4A). This is in agreement with our disulfide mapping results. Cys3, Cys7 and Cys11 are located in the N terminal loop, which is stabilized *via* disulfide bonds with Cys26, Cys31 and Cys30 of the π helix. Cys14 is situated at the C-terminus of the one-turn 3_10_ helix and connects with Cys25 of the π helix (molecule A) and α helix (molecules B and C). These four disulfide bonds enable the short peptide lyba2 to form a compact and constrained structure.

The interactions between molecules A, B, and C, as well as their symmetry-related copies, form the lyba2 crystal lattice. Interestingly, we found that a short polyethylene glycol molecule (PEG, HO-(CH_2_-CH_2_-O)_3_-CH_2_-CH_2_-OH, here named “P40”) stabilizes the crystal lattice by filling a cleft between molecule C and its symmetry-related copy C* (symmetry operator: X-1, Y, Z; Fig. 4B). The PEG molecule is well defined by electron density (Fig. 4C). Its torsion angles about the C-O bonds are in the antiperiplanar range and those about the C-C bonds are in the synclinal range, as is common for polyethers^12^. The majority of its interactions with peptide copies C and C* consist of water-mediated hydrogen bonds (Fig. 4C).

### Metabolic stability of lybatides

**Figure 5:**
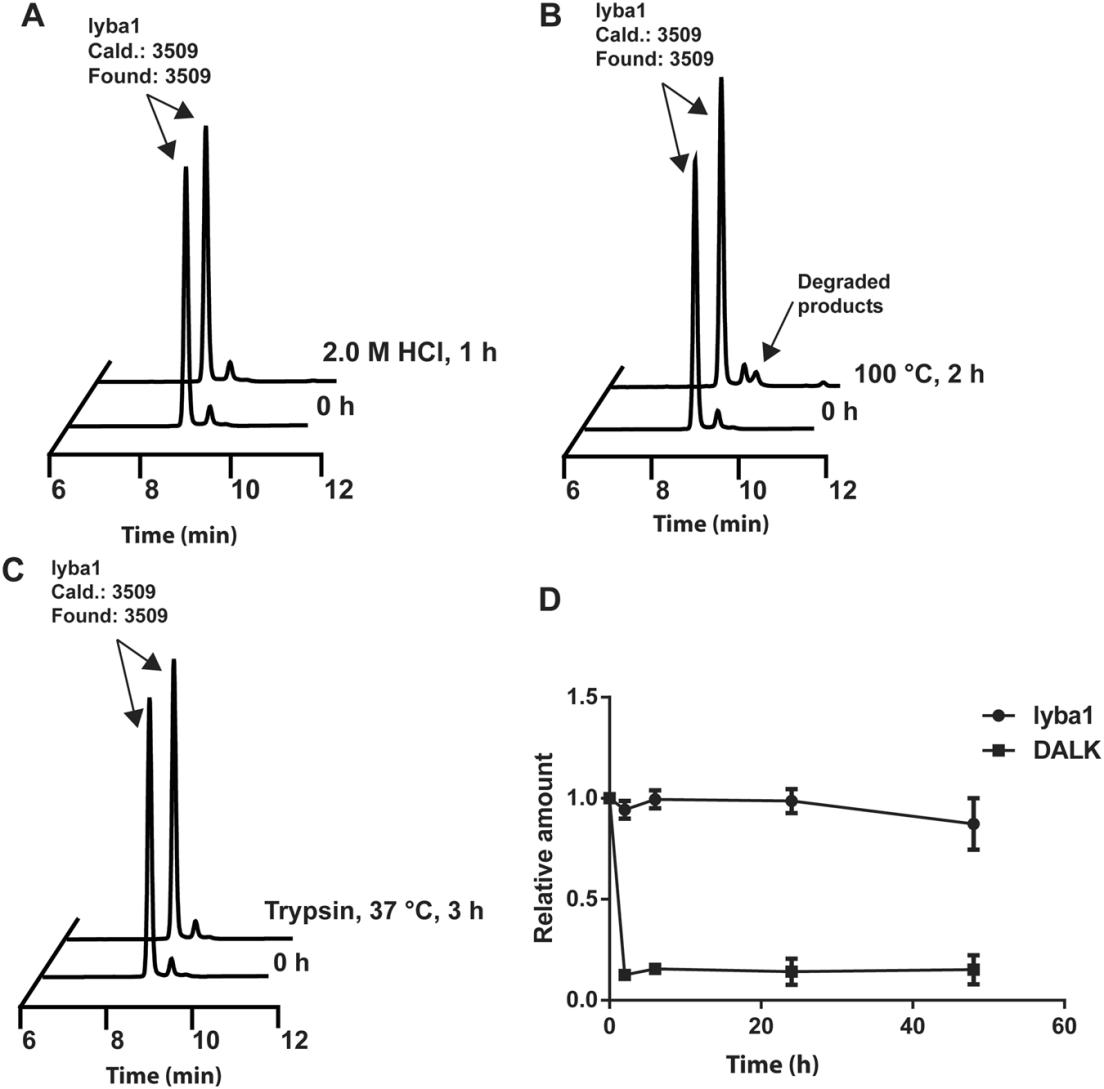
Stability assays of lybatide, lyba1. (**A**) Thermal stability of lyba1 incubated at 100 °C for 2 h. (**B**) Acidic condition stability of lyba1 incubated in 2.0 M HCl (pH 2.0) for 1 h. (**C**) Trypsin enzymatic stability of lyba1 incubated for 3 h in buffer as suggest by manufactures at 37 °C. (**D**) Stability of lyba1 incubated in human serum for 60 min at 37 °C, DALK was used as a control. The molecular weight of the peak was determined by MALDI-MS.

CRPs with a well-defined disulfide core are known for their compact structure and high resistance to heat and enzymatic degradation. This stability is an important characteristic for CRPs as putative bioactive peptides in order for them to survive decoction processes in traditional herbal preparations and to retain both bioavailability and pharmacological effects by oral administration. Figure 5 shows that lyba1 exhibits minimal degradation and denaturation in harsh testing conditions. A total of 96% of lyba1 remained after heating in boiling water at 100 °C for 2 h. Similarly, 99% of the peptide remained intact after incubation in 2 M HCl for 3 h, and 99% of the peptide was not degraded when incubated with trypsin for 3 h (Fig. 5).

### Anti-bacterial activity and cytotoxicity of lyba1

Many CRPs including defensins, thionins and hevein-like peptides display antimicrobial or cytotoxic activity^6^. To determine whether lybatides possess similar antimicrobial activity, a radial diffusion assay was performed with lyba1 on four different microorganisms. Lyba1 did not show any antimicrobial activity against these specific strains when tested up to 1 mM. MTT assays were performed to evaluate the cytotoxic effect of lyba1 on three different cell lines which lyba1 did not display any cytotoxic effect when tested up to 100 μM (Fig. S3).

## Discussion

In the present study, we report the isolation and characterization of two novel helical CRPs, lyba1 and lyba2, from the cortex of *L*. *barbarum* roots. Lybatides are cysteine-rich and highly constrained peptides. They show no sequence similarity to any known plant CRPs. More importantly, they display a unique cysteine framework (Table 1) and a novel structure containing a dominant α-helix that is cystine-stapled and not stabilized by another β-sheet or another helix. The small of size lybatides compared to defensins and thionins plus the presence of a cystine-stapled α-helix suggests that lybatides represent a new CRP family.

**Table 1:** Sequence alignment of plant CRPs from different CRP families.

Family	Peptide	No. of SS	Amino Acid Sequence	Ref.
Lybatides	lyba1	4	-DSCSE--------YCSNNSCP-YC--DGQ--KLYTLC---C-IN--TCCPS------	This work
	lyba2	4	-DSCSE--------YCSN-RCP-SC--DGQTQTQYTLC---C-IN--ICCPS------	This work
Defensins	NaD1	4	-RECKTESNTFPGICITKPPCRKAC---ISEKFTDGHC---SKILRRCLCTKPC----	32
α-hairpinin	Ec-Amp	2	--GSGR--------GSCRSQCMRR---HEDEPWRVQEC-----VS---QCRRRRGGGD	58
Thionins	β-Purothionin	4	-KSCCK--------STLGRNCYNLCRARGAQKLCANVCR--CKLTSGLSCPKDFPK--	18
Jasmintides	jS1	3	-QLCLQ--------CRSNSDCN----------IIWRIC-----RDG----CCNVI-----	15
Knottins	cT1	3	-DPTCS--------VLGDFKCN-----PGRCCSKFNYCGSTAAYCGPGNCIAQCP---	60
Hevein-like peptides	gB5	4	GIPCGE--------SCVFIPCI----------TGAIGC-----SCKSKVCYRN-----	36

Mass spectrometric sequencing of lybatides revealed that they contain eight cysteines (8C-CRPs) with a distinctive C-C-C-C-CC-CC cysteine arrangement. To the best of our knowledge, the presence of two consecutive CC motifs at the C-terminus of lybatides has not been reported for plant CRPs. The CC motif, with two adjacent cysteine residues and no amino acid in between, is often a diagnostic element in CRP families (Table 1). For example, thionins contain a CC motif at their N-terminus, displaying a cysteine spacing of CC-C-C-C-C-C-C, whereas trans-defensins contain a CC motif at the C-terminus as C-C-C-C-C-C-CC^9^. The presence of the CC motif in the middle can be found in CRPs such as cystine-knot α-amylase inhibitors, hevein-like peptides and jasmintides^13–15^.

Disulfide mapping of lybatides revealed a novel disulfide connectivity of CysI‒CysVI, CysII‒CysVIII, CysIII‒CysVII and CysIV‒CysV. Currently, only three general types of disulfide connectivity are known in plant CRPs. Of the three, the most recently discovered disulfide connectivity, CysI–V, CysII–IV and CysIII–VI, is found in jasmintides^15^. The remaining two are cystine knots represented by defensins, knottins^16^ or hevein-like peptides^17^ and symmetrics represented by thionins^18^ and α-hairpinins^19^. Between these two, cystine knot motif is by far the more common type of disulfide connectivity in plant CRPs. Lybatides appear to be a hydrid of cystine knot and a symmetric (Table S2). Upon the removal of the CysI‒VI disulfide bond in lybatides, the remaining disulfides, CysII‒VIII, CysIII‒VII and CysIV‒V, result in the arrangement of a symmetric^20, 21^. However, plant defensins and thionins are generally basic and hydrophobic, whereas lybatides are anionic and hydrophilic.

The presence of negatively charged phospholipids and negatively charged structures, such as teichoic acid in Gram-positive and lipopolysaccharides in Gram-negative bacteria, at the membranes of bacteria results in a negatively charged environment. This would allow positively charged antimicrobial peptides like plant defensins and thionins to attach to the plasma membrane^22^. In addition, the hydrophobic nature of cysteine-rich petides would allow them to interact with the lipid bilayer and thus disrupt the integrity of the membrane resulting in cell death^23^. The anionic and hydrophilic nature of lybatides would prevent lybatide from interacting with the negatively charged bacterial membrane of the bacteria. This may explain why lybatides do not display the cytotoxic and antimicrobial effects that are commonly displayed by thionins and plant defensins.

Sharing the same cysteine framework as lybatides is the conotoxin RsXXIVA, a Ca_v_2.2 channel inhibitor isolated from the venom duct of *Conus regularis* ^24^. However, they only share a sequence similarity of 46.9%. Since cysteine scaffolds have been known to tolerate a hypervariable amino acid sequences while maintaining similar overall structure, thus it is likely lybatides and RsXXIVA would share similar disulfide connectivities and structural fold. As the structure of conotoxin RsXXIVA has not been reported, our X-ray crystallographic and disulfide connectivity studies may be helpful in predicting the structure of RsXXIVA.

Analysis of the solid-state tertiary structure of lyba2 revealed an (α + π)-helix stabilized by three disulfide bonds as a dominant structural feature. A cystine-stabilized (α + π)-helix is rarely seen in small disulfide-rich peptides. An α-helix involved in disulfide bonds is commonly observed in CRP families such as thionins and plant defensins^25^. Two commonly known motifs of cystine-stabilized α-helix structures are the CSαα and CSαβ motif. The CSαα motif is found in thionins and α-hairpinins, and is distinguished by an α-helix stabilized by another α-helix, with disulfide bonds cross-bracing between the two α-helices. In contrast, the CSαβ motif is found in plant defensins and contains a β-sheet to stabilize an α-helix, with disulfide bonds connecting the α-helix to the central β-strand to reinforce the CSαβ structure^26^.

**Figure 6:**
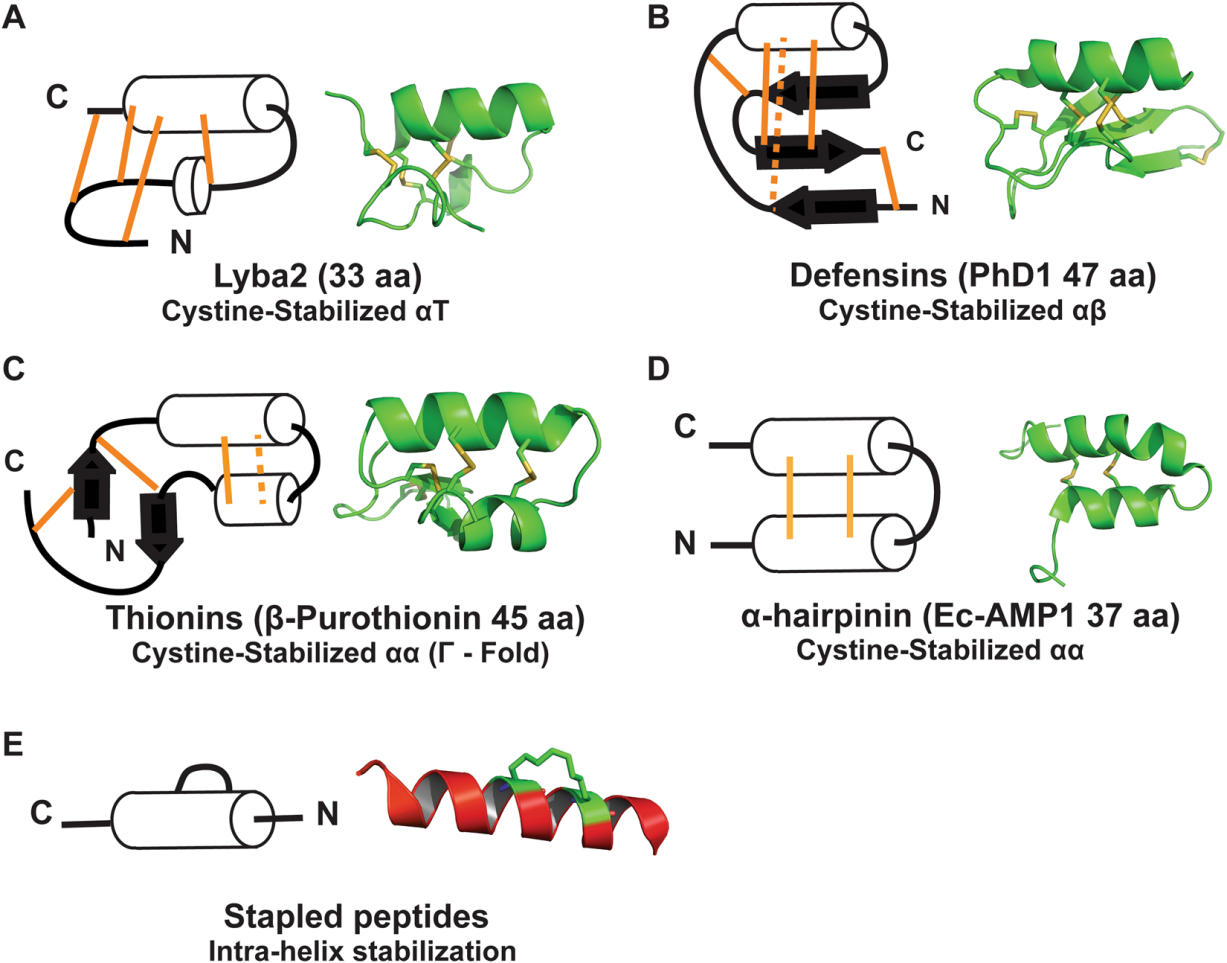
Cartoon and 3D structures of lybatide lyba2, defensin PhD1, thionin β-purothionin, α-hairpinin Ec-amp and stapled peptides. (**A**) Structure of lyba2. The N-terminal half of the peptide loops back along the (α + π)-helix stabilizing it through hydrogen and disulfide bonds. (**B**) The structure of plant defensin contains a β1-loop-α-β2-β3 pattern in the secondary structure, resulting in the CSαβ motif where the α-helix is stabilized by the anti-parallel β-sheet through disulfide bonds connecting the α-helix to the center strand of the β-sheet^57^. (**C**) The overall structure of thionins can be represented as by the Greek capital letter Г. The long arm is made up of antiparallel α-helices and the short arm contains a region of extended conformation and a short anti-parallel β-sheet. Disulfide crosslinking occurs within the two arms separately^18^. (**D**) The two helices in α-hairpinins are stabilized by two parallel disulfide bonds^58^. (**E**) The stabilization of the helical structure of stapled peptides occurs within the helix and is stabilized by hydrocarbon chains^59^. (PDB ID: (**B**) 1N4N, (**C**) 1BHP, (**D**) 2L2R, (**E**) 4MZK).

Figure 6 shows five different types of α-helical peptides. Of the five, the most well-known is the CSαβ structural motif which is found in plant defensins (Fig. 6B). It is composed of one α-helix and three antiparallel β-strands^27^. The CSαβ motif was first characterized in charybdotoxin^28, 29^. This motif contains a β1-loop-α-β2-β3 pattern in the secondary structure, where the α-helix is parallel to three antiparallel β-strands^30^. The α-helix is stabilized by two disulfide bridges connected to the central β-strand of the antiparallel β-sheet^31^. CSαβ peptides such as plant defensins are also characterized by the presence of a cystine knot, CysII‒V, CysIII‒VI and CysIV‒VII, with an extra disulfide bond, CysI‒VIII, in the structure (based on NaD1, a plant defensin isolated from *Nicotiana alata*)^32^. The second and third types display a CSαα structural motif which is found in thionins and α-hairpinins (Fig. 6C and D). In thionins, the CSαα structural motif is composed of two α-helices and two anti-parallel β-strands. This motif contains a β1-α1-α2-β2-loop pattern^21^. The α-helices are stabilized by disulfide bonds cross bracing between each other, with no disulfide bonds connecting the α-helix to either β-strand. In α-hairpinins, their helical conformation is usually stabilized by another α-helix. The fifth type is the all-hydrocarbon-stapled helical peptide (Fig. 6E), which is not found in nature and will be further elaborated below.

Instead of the α-helix – α-helix cystine stabilization found in thionins or the α-helix – β-sheets stabilization in plant defensins, the (α + π)-helix in lybatides is stabilized by four disulfide bonds located in two turns (the β-turn 2–6 and the 3_10_-helical turn 12–14) and one loop (7–11) in the N-terminal portion of the peptide. In addition to these four disulfide bonds, the main-chain amide of Cys3 (located in turn 2–6) forms a hydrogen bond with the side-chain of Tyr22 in the α-helix. The side-chain amide nitrogen of Asn9 (located in the 7–11 loop) donates a hydrogen bond to the main-chain C=O of Cys30 (in the π-helix). Compared to thionins, lybatides exhibit a fold that is similar but in an opposite direction, resulting in a structure that resembles a capital “L” rather than the Г fold displayed by thionins. Comparison of the structures of lybatides and certain members of the thionins and plants defensins with TM-align^33^ gives a TM score of 0.36–0.39 and 0.33–0.36, respectively (Table S3) (where a score of 0.5 indicates similar structural fold). The difference in structural features, when compared to other cystine-stabilized α-helical peptides, together with the presence of a α-helix in lybatide would classify lybatides as a new family of the cystine-stabilized α-helical peptides.

The distortion of the α-helix towards a π-helix in the C-terminal half of lybatides is of interest. This is very likely caused by conformational stress induced by the disulfide bonds at the C-terminus (Cys30 and Cys31). The π-helix conformation is postulated to be unfavorable and thus would tend to be selected against unless associated with a functional advantage^34^. This suggests that the active functional site of lybatides may be located at the region where the π-helix is found, as the π-helix has been shown to play a functional role within certain protein binding sites^35^.

### Biosynthetic pathway of lybatides

**Figure 7:**
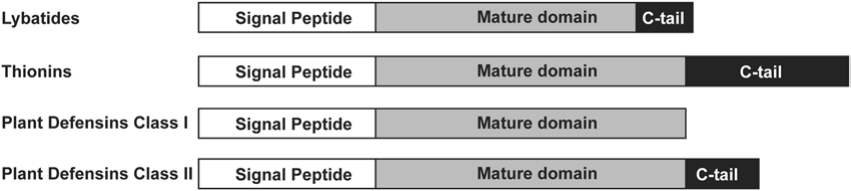
Precursor gene structure organization of lybatides. (**A**) Predicted precursor structure of lybatides based on homologous sequences. A signal peptide followed by the mature domain and a C-tail of about 12–15 amino acids. (**B**) Precursor structure of thionins, where the mature domain is flanked by the signal peptide and an acidic C-tail. (**C**) Precursor structure of the plant class I defensins. They do not contain the C-tail domain (**D**) Precursor structure of the plant C8 class II defensins. They have a similar three-domain organization as lybatides and thionins.

A sequence query using t-BLASTn with lybatide on the oneKP transcriptomic database (www.oneKP.com) revealed two homologous sequences with 78.8% (OSMU-2070462) and 69.7% (OSMU-2073250) similarity to lyba2. The homologous sequences suggest that the precursor of lyba1 and lyba2 contain a three-domain arrangement: a signal peptide followed by the mature domain and a C-terminal tail of about 12–15 amino acids (Fig. 7). This precursor organization is similar to the one found in thionins, where the mature domain is flanked by the signal peptide and an acidic C-terminal tail^6^. The three-domain precursor arrangement is commonly observed in plant CRPs including cystine-knot α-amylase inhibitors, hevein-like peptides and knottins^36, 37^. However, in defensins, there are two types of precursor structure organization. The plant C8 class II defensins have a similar three domain organization as lybatides and thionins, whereas the plant class I defensin precursors do not contain the C-tail domain^27^.

### Lybatide as a helical peptide-displayed scaffold

Naturally occurring constrained peptides generally have shapes that have evolved to fit precisely into the binding pockets of their targets in addition to contributing to their stability, and thus, they provide excellent scaffolds for peptide drug design and engineering^7^. The presence of a three-turn (α + π)-helix in lybatides is potentially useful for peptide engineering. An α-helix is a common structural motif found in many bioactive peptides. The development of chemical methods to stabilize bioactive conformations remains an active area of research^38^. Approaches aimed at reinforcing α-helical conformations include constraints by disulfide bonds^39^, lactam bridges^40^ and all-hydrocarbon crosslinks to create stapled peptides^41^ (Fig. 6E). An increase in helical structure often leads to enhanced target-binding and metabolic stability. In certain cases of stapled peptides, it has also been shown that it leads to membrane permeability to interrupt protein-protein interactions. In this regard, the cystine-stabilized peptides may be considered naturally-occurring “stapled peptides”. However, there is a subtle distinction. The synthetic all-hydrocarbon stapled peptides employ intra-helix stapling of side chains of a helical strand, whereas cystine-stapled helical lybatides use inter-strand disulfide bonds to stabilize the helical conformation.

The discovery of a new cystine scaffold from nature holds promise for its use in protein engineering. Pease *et al*.^25^ demonstrated that the activity of a particular peptide fold will not necessarily be affected by disulfides used for stabilization, provided that the contact surface is correctly exposed and that the stabilized secondary structure is the correct one. As cysteine-rich peptides are evolvable and can tolerate a wide range of sequences^42^, this allows them to serve as scaffolds to increase stability in designing peptidyl drugs. Vita *et al*. provided an early example two decades ago by grafting a nonapeptide onto the charybdotoxin, a scorpion toxin, scaffold resulting in a highly potent Cu^2+^-binding peptide^43^. Since then, numerous examples of successful grafting of bioactive peptides to cysteine-rich rich scaffolds have been reported^44–48^. The high mutational tolerance of cysteine-rich peptides allowed Bhardwaj *et al*. to introduce de novo designed scaffolds without impairing folding and solubility^7^. Thus it is logical to envision that the lybatide scaffold is suitable for grafting and mutational engineering of peptides and proteins. Of particular interest is the presence of the π-helix in the lybatide scaffold which may expand the peptide drug engineering repertoire, especially where a π-helix could be more suitable than the conventional α-helix for binding to the target protein^41^.

## Conclusion

Lybatides display a novel structural motif, along with a novel disulfide connectivity, which has not been reported in plant CRPs with MW <4 kDa. The discovery of lybatides with a novel cystine motif expands the number of different classes of CRPs that are known to occur in nature. These findings expand the diversity of disulfide connectivity in peptides with four to ten cysteine residues. An important finding of this work is that lybatides are a new addition to the current repertoire of CRP-based structures and could serve as a cystine-stabilized helical template for drug design^44^.

## Experimental Procedures

### Extraction and screening

Dried cortex of *L*. *barbarum* root was purchased from a local distributor (Hung Soon Pte Ltd, Singapore)^49^. The sample was extracted with the appropriate extraction solvent (water, 50% EtOH) in a 1:10 ratio (v/v) at room temperature. The extracts were then centrifuged at 3000 × *g* for 30 min to remove plant debris. ZipTip C_18_ (Millipore) was used for sample preparation of the crude extracts for mass spectrometry analysis. Mass spectra of the samples were obtained with a 4800 Proteomics Analyzer MALDI-TOF/TOF mass spectrometer (Applied Biosystems, California, USA). The MS spectra were acquired using reflector and/or linear mode, using up to 2500 shots and an accelerating voltage of 20 kV.

### Isolation and purification of CRPs

Dried *L*. *barbarum* root cortex (500 g) was extracted with 500 mL of distilled water. The aqueous extract was fractionated by flash chromatography with C_18_ beads followed by preparative HPLC using a C_18_ Vydac column (250 × 21 mm) on a Shimadzu HPLC system at a flow rate of 5 mL/min. A linear gradient of 1% per min of 5–80% buffer B was applied using buffer A (0.1% v/v trifluoroacetic acid; TFA; in Milli-Q water) and buffer B (0.1% v/v TFA in acetonitrile). The fractions containing the CRP of interest were purified with a preparative SCX-HPLC with a PolySULFOETHYL A column (particle size: 10 µm, 250 × 21 mm; PolyLC Inc., USA). A linear gradient of 0–60% buffer B was applied (buffer A contained 20% ACN, 10 mM NaH_2_PO_4_, pH 2.8; buffer B contained 20% ACN, 1.0 mM NaCl, 10 mM NaH_2_PO_4_, pH 2.8). Further purification was performed on a semi-preparative C_18_ Vydac column (particle size: 10 µm, 250 × 10 mm; Grace, Maryland, USA) at a flow rate of 3 mL/min with the same gradient^50^.

### Sequence determination

Lybatides lyba1 and lyba2 (10 μg) were dissolved in 30 μL of 100 mM ammonium bicarbonate buffer (pH 7.8) containing 10 mM dithiothreitol (DTT) and incubated for 2 h at 37 °C. The *S*-reduced peptides were either *S*-alkylated first with 4-vinylpyridine followed by enzymatic digestion or directly digested with a trypsin or chymotrypsin (Roche, Basel, Switzerland) solution at 1 μg/μL with a final peptide-to-enzyme ratio of 50:1 and incubated for 30 min. The peptide fragments obtained were then examined by MALDI-TOF MS followed by MS/MS analysis in positive ionization mode. The MS and MS/MS spectra were acquired using a dual-stage reflectron mirror and up to 2500 shots in MS and 5000 shots in MS/MS mode. Nitrogen gas was used as the collision gas, and a collision energy of 1 keV was used in collision-induced dissociation. The MS/MS spectra were then analyzed, and the sequences of the amino acids were determined based on the presence of b- and y-series ions. Assignments of isobaric residues Lys/Gln and Leu/Ile were based on sequence comparison to known homologs or by comparison with transcriptome data from the OneKP database (http://www.onekp.com).

### Disulfide mapping

Lyba1 was partially reduced in 100 µL of 50 mM Tris(2-carboxyethyl)phosphine (TCEP) and 10% ACN at 65 °C for 2 min. Subsequently, N-ethylmaleimide (NEM) was added to a final concentration of 250 mM and incubated at 37 °C for 1 h. The intermediates were then fractionated with an analytical RP-HPLC with a Vydac C_18_ column (particle size: 5 µm, 250 × 4.6 mm; Grace, Maryland, USA) at a flow rate of 0.3 mL/min with a gradient of 23–37% over 80 min with buffer A (0.1% TFA) and buffer B (100% ACN, 0.1% TFA). Intermediate species were collected and analyzed using MALDI-TOF MS. Each intermediate species was then fully reduced with 50 µL of 20 mM DTT, 25 mM ammonium bicarbonate in 20% (v/v) ACN at 37 °C for 1 h and alkylated with 40 mM iodoacetamide (IAA) at 37 °C for 2 h. Alkylated samples were then examined by MALDI-TOF/TOF and sequenced by tandem mass spectrometry.

### X-ray crystallography of lyba2

Purified lyba2 was dissolved in deionized water (10 mg/mL). This sample was then used to perform crystallization experiments at 18 °C with a Phoenix crystallization robot (Art Robbins, USA) employing the sitting-drop vapor-diffusion method, 0.3 µL of peptide and 0.3 µL of reservoir were mixed and equilibrated against 75 µL reservoir solution. Two commercially available screens were used: Index^TM^ (Hampton Research, USA) and Structure Screen1/2 MD1–01/02 (Molecular Dimensions, United Kingdom). Tiny crystals of lyba2 were observed under condition No. 30 of MD1–01 (2.0 M ammonium sulfate, 0.1 M sodium HEPES pH 7.5, 2% PEG400). Optimized crystals were subsequently obtained within three months from 1.6 M ammonium sulfate, 0.1 M sodium citrate tribasic dihydrate, pH 4.5, 2% PEG400, with mixing 2 µL of peptide and 2 µL of reservoir to equilibrate against 500 µL reservoir.

Optimized crystals were cryo-protected with 20% glycerol and flash-cooled in liquid nitrogen. A native dataset to 1.48-Å resolution was collected at a wavelength of 0.9184 Å, and a sulfur single-wavelength anomalous dispersion (S-SAD) dataset to 1.95 Å was collected at a wavelength of 1.8000 Å, both at beamline P11 of the PETRA III storage ring (DESY, Hamburg, Germany). Data collection followed the recommendations for S-SAD phasing described by Weiss *et al*.^11^. Both diffraction datasets were processed with XDS^51^. The space group was P2_1_, with unit-cell parameters *a* = 19.32 Å, *b* = 54.65 Å, *c* = 42.40 Å, and β = 100.60°. The structure of lyba2 was determined by the SAD method. Twenty of 24 sulfur atoms were found using SHELXD^52^. The correct hand for the substructure was determined and a preliminary poly(Ala) chain was modeled with SHELXE^52^. This initial model was then rebuilt and refined using Coot^53^ and REFMAC 5^54^. The refined structure was visualized using Pymol (Schrödinger; http://www.pymol.org/) and UCSF Chimera^55^.

### Stability Studies

Heat stability: Lybatide lyba1 (10 μg) was added to 100 μL of distilled water and incubated at 100 °C for 1 h. A replicate at room temperature was done as a control. The RP-HPLC profiles of the heated and the control sample were compared to evaluate their stability.

Acid stability: Lybatide lyba1 (10 μg) was added to 100 μL of 2.0 M HCl and incubated at 37 °C for 1 h. A replicate was done as a control in deionized water. The RP-HPLC profiles of the reaction sample and the control sample were compared to evaluate their stability.

Enzymatic stability: Lybatide lyba1 (10 μg) was added to 100 μL of 100 mM ammonium bicarbonate buffer (pH 7.8). Next, 1 μL of 0.5 μg/μL trypsin was added and incubated at 37 °C for 3 h. A replicate was done as a control without adding trypsin. The RP-HPLC profiles of the treated and the control sample were compared to evaluate their stability.

Human serum stability: Lybatide lyba1 (0.1 M) was prepared in 25% human serum in DMEM medium without phenol red. The test samples were incubated at 37 °C. DALK was used as a positive control. Samples were collected at various time points (0, 1, 2, 24 and 48 h). The collected samples were subjected to protein precipitation with 100% ethanol and centrifugation at 180,000 × *g* for 5 min at 4 °C. The supernatant was collected for analysis.

### Cell Toxicity assay

Lybatide lyba1 was tested for its cytotoxicity effect against human umbilical vein endothelial cells (HUVEC-CS, ATCC CRL-2873), monkey kidney epithelial cells (Vero, ATCC CCL-81) and adenocarcinomic human alveolar basal epithelial cells (A549, ATCC CCL-185). Briefly, 1 × 10^4^ cells per well in 100 μL were seeded into a 96-well plate (TPP, Switzerland) and incubated overnight. The supernatant was removed, and the cells were washed twice with phosphate buffered saline (PBS; Gibco, USA). The cells were then treated with lyba1 at concentrations 0.01 μM, 0.1 μM, 1 μM, and 10 μM and incubated overnight, with 10% DMSO used as positive control.

After incubation, MTT was added to the wells and the plate was incubated for 1.5 h at 36.7 °C. Next, 200 μL of 100% DMSO was added, incubated for 2 h, and the absorbance was determined with a microplate reader at 550 nm wavelength.

### Radial diffusion assay

One Gram-positive bacterial strain (*Staphylococcus aureus*), one Gram-negative bacterial strain (*Escherichia coli*) and two fungal strains (*Candida albicans* and *Candida tropicalis*) were each cultured in tryptic soy broth (TSB) at 37 °C. The anti-microbial activity of lyba1 was examined with the radial diffusion assay as described by Lehrer *et al*.^56^. Briefly, the yeast phase of the fungal bacterial strains was washed with 10 mL sterile cold 10 mM sodium phosphate buffer (pH 7.4) and resuspended in sterile cold sodium phosphate buffer (5 mL). The optical density of the subcultures was measured to determine the concentration of cells. Next, 4 × 10^6^ CFU was added to 10 mL of molten underlay gel containing 10 mM NaCl and poured into 60 mm × 15 mm petri dishes. Then, 3.2-mm-diameter wells were punched into the underlay gel in a 4 × 3 array, and 5 µL of lyba1 solutions at different concentrations was added to each well. After the addition of peptide, the plates were inverted and incubated for 3 hours at 37 °C. A total of 10 mL of molten overlay gel was then poured above the underlay gel, and the plates were incubated overnight. The diameters of the clear zones around each well were measured.

## Electronic supplementary material

Supplementary Information
