# Supplementary material for: Lybatides from Lycium barbarum Contain An Unusual Cystine-stapled Helical Peptide Scaffold
Source: Sci Rep. 2017 Jul 12;7:5194. doi: 10.1038/s41598-017-05037-1 (PMC5507927; doi:10.1038/s41598-017-05037-1)
Supplement: Supplementary file 1 — Supplementary Information [file 41598_2017_5037_MOESM1_ESM.pdf]

Lybatides from *Lycium barbarum* Contain An Unusual Cystine-stapled Helical Peptide Scaffold

Authors

Wei Liang Tan[1], Ka H. Wong[1], Jian Lei[2,3], Naoki Sakai[2], Hong Wei Tan[1], Rolf Hilgenfeld[2,3], James P. Tam[1*]

Affiliations

[1]School of Biological Sciences, Nanyang Technological University, Singapore

[2]Institute of Biochemistry, Center for Structural and Cell Biology in Medicine, University of Lübeck, Ratzeburger Allee 160, 23562 Lübeck, Germany

[3]German Center for Infection Research (DZIF), Hamburg–Lübeck–Borstel–Riems Site, University of Lübeck, Germany

Supplemental Information

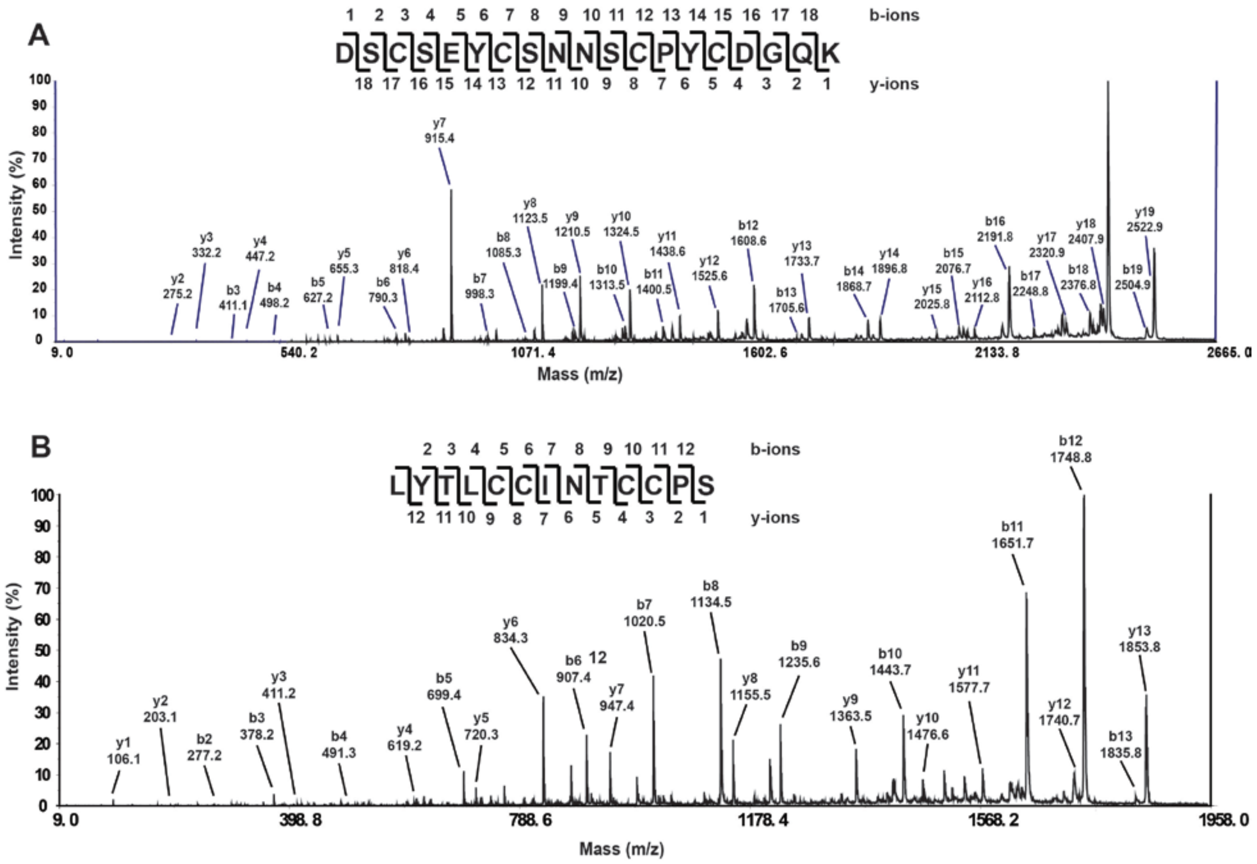

Figure S1. *De novo* sequencing of lyba1. Tryptic digestion of *S*-alkylated lyba1 produced two fragment, one with m/z of 1853 (A) and another with m/z of 2523 (B). Tandem mass spectrometry was used to determine the primary peptide sequence as DSCSEYCSNNSCPYCDGQKLYTLCCINTCCPS.

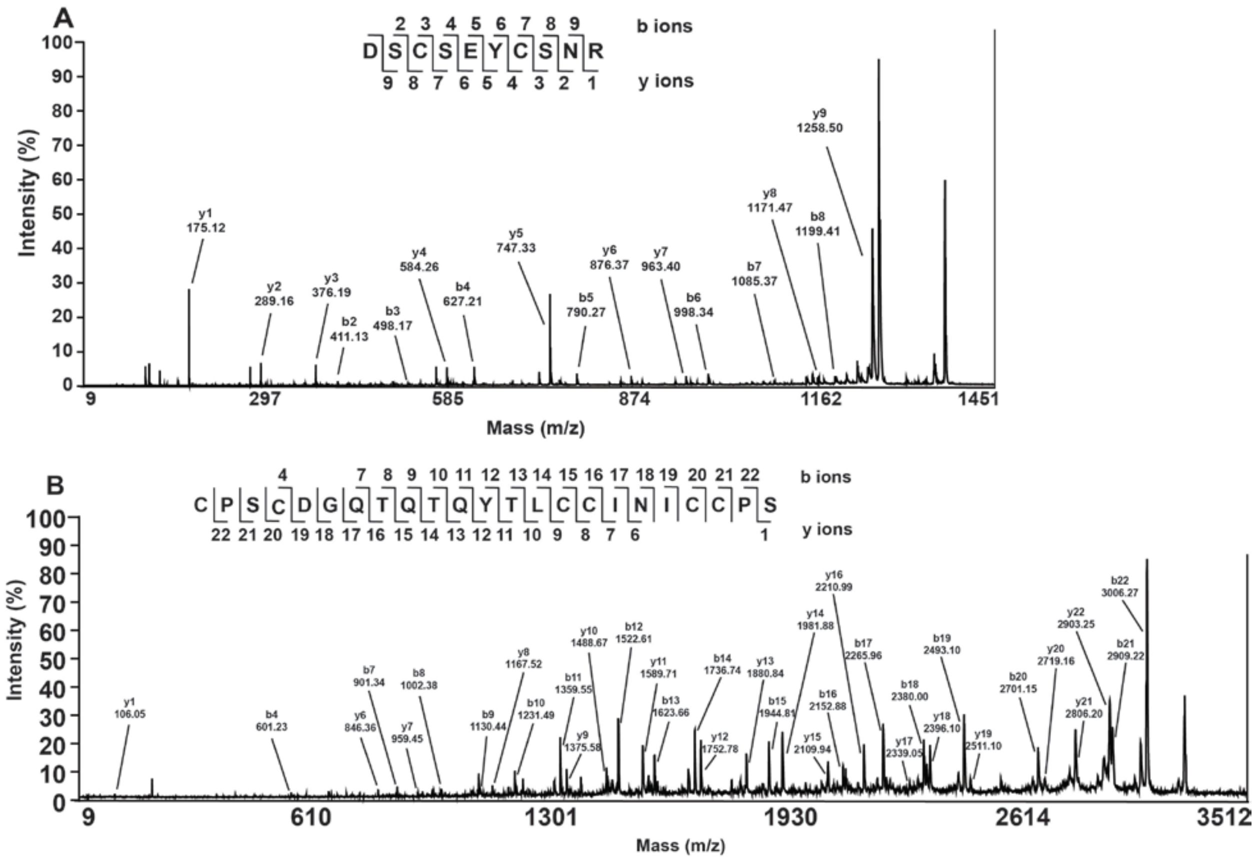

Figure S2. *De novo* sequencing of lyba2. Tryptic digestion of *S*-alkylated lyba2 produced two fragment, one with m/z of 1372 (A) and another with m/z of 3109 (B). Tandem mass spectrometry was used to determine the primary peptide sequence as DSCSEYCSNRCPSCDGQTQTQYTLCCINICCPS.

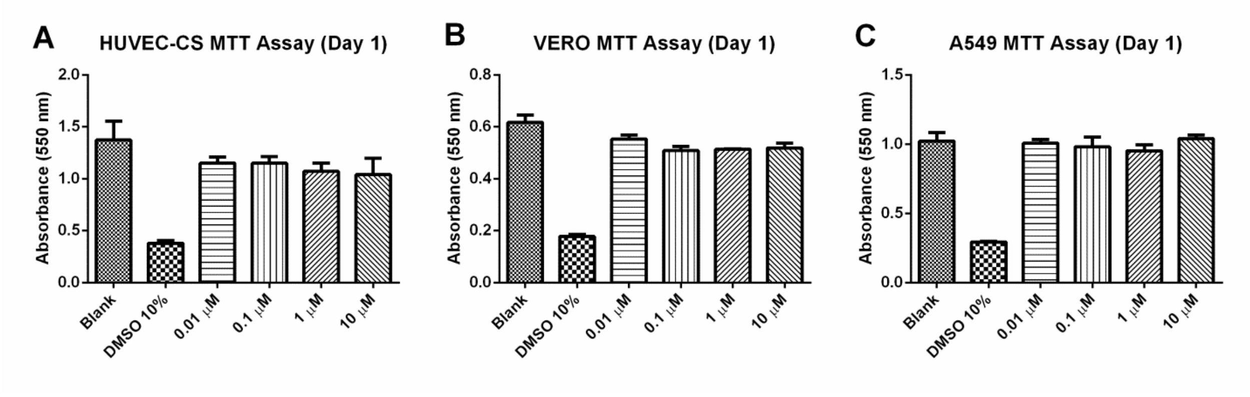

Figure S3. MTT assay of lybatides on (A) human embilical vein cord cells (HUVEC-CS), adenocarcinomic human alveolar basal epithelial cells (A549) and monkey kidney epithelial cells (vero). 10% DMSO were used as control. No significant difference were detect after one day of incubation with lyba1 from testing concentrations of up to 10 μM as compared to blank.

Table S1. Data collection and refinement statistics for lyba2

	Diffraction data	
	Native	Sulfur-SAD
Data collection statistics		
Space group	$P2_1$	$P2_1$
Unit-cell dimensions (Å, °)		
	a = 19.32, *b* = 54.65	*a* = 19.37, *b* = 54.76,
	c = 42.40	*c* =42.49
	β = 100.60	β = 100.59
Wavelength (Å)	0.9184	1.8000
V_m ($Å^3$/Da)	2.04	2.04
Solvent content (%)	39.8	39.8
Resolution range (Å)	41.68-1.48 (1.56-1.48)	41.77-1.95 (2.06-1.95)
Number of unique reflections	14465 (2057)	6382 (879)
R_{merge}[1]	0.033 (0.039)	0.037 (0.203)
R_{pim}[2]	0.014 (0.017)	0.023 (0.173)
Completeness (%)	99.6 (98.4)	98.8 (96.5)
Mean I/σ(I)	44.3 (31.8)	9.7 (7.0)
$CC_{1/2}$[3]	0.999 (0.998)	0.997 (0.822)
Multiplicity	6.5 (6.3)	5.8 (3.9)
Wilson B-factor ($Å^2$)	4.2	14.2
Refinement statistics		
R_{cryst} (%)[4]	10.76	
R_{free} (%)[4]	13.70	
Protein atoms	731	
Water molecules	155	
Ligands	5	
Clashscore[5]	4	
r.m.s. deviation in bond lengths (Å)	0.02	
r.m.s. deviation in bond angles (°)	1.95	
Average *B*-factor for all atoms ($Å^2$)	10.6	
Ramachandran plot		
Residues in favored regions (%)	91.2	
Residues in additionally allowed regions (%)	8.8	
Residues in outlier regions (%)	0	

[1] $R_{merge} = \sum_{hkl}\sum_i | I_i(hkl) - \langle I(hkl)\rangle | / \sum_{hkl}\sum_i I_i(hkl)$ [1].

[2] $R_{pim} = \sum_{hkl} \{1/[N(hkl) - 1]\}^{1/2} \times \sum_j | I_i(hkl) - \langle I(hkl)\rangle | / \sum_{hkl}\sum_j I_i(hkl)$ [2].

[3] $CC_{1/2}$ is the correlation coefficient determined by two random half data sets [3].

[4] $R_{cryst} = \sum_{hkl} | F_o(hkl) - F_c(hkl) | / \sum_{hkl} F_o(hkl)$. R_{free} was calculated for a test set of reflections (4.9%) omitted from the refinement.

[5] Clashscore is defined as the number of clashes calculated for the model per 1000 atoms (including hydrogens) of the model. Hydrogens were added by *MolProbity* [4]

.

Table S2. Major families of plant cystine-rich peptides

Peptide family	S-S No.	Representative member			
		Name	AA No.	Disulfide motif	References
Knottins	6	PCI	39	C-C-C-C-C-C	[18]
Defensins	4	NaD1	47	C-C-C-C-C-C-C-C	[32]
α-Hairpin	2	Ec-AMP1	37	C-C-C-C	[50]
Thionins	4	β-Purothionin	45	CC-C-C-C-C-C-C	[20]
Jasmintide	3	jS1	27	C-C-C-C-CC	[17]
Lybatides	4	lyba2	33	C-C-C-C-CC-CC	This Work

Table S3. TM-alignment score of cysteine-rich peptides with α-helix in the structure.

Peptide	Family	TM-alignment score
Crambin	Thionins	0.36316
B-purothionin	Thionins	0.39036
Viscotoxin	Thionins	0.37814
NaD1	Defensins	0.35518
PhD1	Defensins	0.33433
VrD1	Defensins	0.35667

References

1 Arndt, U. W., Crowther, R. A. & Mallett, J. F. A computer-linked cathode-ray tube microdensitometer for x-ray crystallography. *J Sci Instrum* 1, 510-516 (1968).

2 Weiss, M. S. & Hilgenfeld, R. On the use of the merging R factor as a quality indicator for X-ray data. *J Appl Crystallogr* 30, 203-205, doi:Doi 10.1107/S0021889897003907 (1997).

3 Karplus, P. A. & Diederichs, K. Linking Crystallographic Model and Data Quality. *Science* 336, 1030-1033, doi:10.1126/science.1218231 (2012).

4 Chen, V. B. *et al.* MolProbity: all-atom structure validation for macromolecular crystallography. *Acta Crystallogr D Biol Crystallogr* 66, 12-21, doi:10.1107/S0907444909042073 (2010).
